# World Vegetable Center Eggplant Collection: Origin, Composition, Seed Dissemination and Utilization in Breeding

**DOI:** 10.3389/fpls.2017.01484

**Published:** 2017-08-25

**Authors:** Dalia Taher, Svein Ø. Solberg, Jaime Prohens, Yu-yu Chou, Mohamed Rakha, Tien-hor Wu

**Affiliations:** ^1^World Vegetable Center Tainan, Taiwan; ^2^Vegetable Crops Research Department, Agriculture Research Center, Horticulture Research Institute Giza, Egypt; ^3^Faculty of Applied Ecology and Agricultural Sciences, Inland Norway University of Applied Sciences Elverum, Norway; ^4^Instituto de Conservación y Mejora de la Agrodiversidad Valenciana, Universitat Politècnica de València Valencia, Spain; ^5^Horticulture Department, Faculty of Agriculture, University of Kafrelsheikh Kafr El-Sheikh, Egypt

**Keywords:** conservation, crop wild relatives, diversity, plant genetic resources, *Solanum melongena*, *Solanum aethiopicum*, *Solanum macrocarpon*

## Abstract

Eggplant is the fifth most economically important solanaceous crop after potato, tomato, pepper, and tobacco. Apart from the well-known brinjal eggplant (*Solanum melongena* L.), two other under-utilized eggplant species, the scarlet eggplant (*S. aethiopicum* L.) and the gboma eggplant (*S. macrocarpon* L.) are also cultivated. The taxonomy and identification of eggplant wild relatives is challenging for breeders due to the large number of related species, but recent phenotypic and genetic data and classification in primary, secondary, and tertiary genepools, as well as information on the domestication process and wild progenitors, facilitates their utilization in breeding. The World Vegetable Center (WorldVeg) holds a large public germplasm collection of eggplant, which includes the three cultivated species and more than 30 eggplant wild relatives, with more than 3,200 accessions collected from 90 countries. Over the last 15 years, more than 10,000 seed samples from the Center's eggplant collection have been shared with public and private sector entities, including other genebanks. An analysis of the global occurrences and genebank holdings of cultivated eggplants and their wild relatives reveals that the WorldVeg genebank holds the world's largest public collection of the three cultivated eggplant species. The composition, seed dissemination and utilization of germplasm from the Center's collection are highlighted. In recent years more than 1,300 accessions of eggplant have been characterized for yield and fruit quality parameters. Further screening for biotic and abiotic stresses in eggplant wild relatives is a priority, as is the need to amass more comprehensive knowledge regarding wild relatives' potential for use in breeding. However, as is the case for many other crops, wild relatives are highly under-represented in the global conservation system of eggplant genetic resources.

## Introduction

Brinjal eggplant (*Solanum melongena* L.) is a warm-weather crop mostly cultivated in tropical and subtropical regions of the world. Two other cultivated eggplant species, the scarlet eggplant (*S. aethiopicum* L.) and the gboma eggplants (*S. macrocarpon* L.), are less known but have local importance in sub-Saharan Africa (Schippers, 2000; Daunay and Hazra, 2012). Based on data from 2014, the global production of eggplant is around 50 million tons annually, with a net value of more than US$10 billion a year, which makes it the fifth most economically important solanaceous crop after potato, tomato, pepper, and tobacco (FAO, 2014). The top five producing countries are China (28.4 million tons; 57% of world's total), India (13.4 million tons; 27% of world's total), Egypt (1.2 million tons), Turkey (0.82 million tons), and Iran (0.75 million tons). In Asia and the Mediterranean, eggplant ranks among the top five most important vegetable crops (Frary et al., 2007).

Regarding nutritional value, eggplant has a very low caloric value and is considered among the healthiest vegetables for its high content of vitamins, minerals and bioactive compounds for human health (Raigón et al., 2008; Plazas et al., 2014b; Docimo et al., 2016). In this respect, eggplant is ranked among the top 10 vegetables in terms of oxygen radical absorbance capacity (Cao et al., 1996). The bioactive properties of eggplant are mostly associated with high content in phenolic compounds (Plazas et al., 2013), which are mostly phenolic acids, particularly chlorogenic acid in the fruit flesh (Stommel et al., 2015) and anthocyanins in the fruit skin (Mennella et al., 2012). Both phenolic acids and anthocyanins have multiple properties beneficial for human health (Plazas et al., 2013; Braga et al., 2016).

Farmers need improved eggplant varieties for sustainable production and adaptation to climate change challenges. Because eggplant has a relatively long growth period, it is more exposed than other vegetable crops to a broad range of plant diseases, pests, nematodes, and weeds. The most common diseases include bacterial wilt, verticillium wilt, fusarium wilt, anthracnose fruit rot, alternaria rot, damping off, Phytophthora blight, phomopsis blight and fruit rot, leaf spot, little leaf of brinjal, and mosaic (Rotino et al., 1997). Eggplant is also subject to attack by numerous insect pests including mites, whiteflies, aphids, eggplant fruit, and shoot borer, leafhopper, thrips, spotted beetles, leaf roller, stem borer, and blister beetle (Rotino et al., 1997; Medakker and Vijayaraghavan, 2007). Unpredictable weather with extreme temperatures, drought or flooding can reduce yield and fruit quality. In general, eggplant breeding programs aim to develop high-yielding varieties, mostly F_1_ hybrids, with high fruit quality, shelf-life and resistance to major disease and insect pests, and with broad adaptation to environmental stress (Daunay and Hazra, 2012).

Access to genetic diversity is fundamental for any breeding program. In this paper, we review the diversity and genetic resources of eggplant. As a point of departure, we examine the taxonomy and relationships of the crop and its wild relatives, as well as the domestication of cultivated eggplant. The relationships among wild, semi-domesticated, and cultivated eggplant are intricate, and the origin, evolution, and migration are incompletely understood (Levin et al., 2006; Meyer et al., 2012). Here, we limit ourselves to identify global occurrences and regions of diversity. A key section is the overview of global genebank holdings of cultivated eggplant and their wild relatives. As we shall demonstrate, for such plants the collection at the WorldVeg is of paramount importance. Composition, seed dissemination and utilization of germplasm from this collection are presented and discussed. The importance of safeguarding and evaluating wild relatives is highlighted, as crop wild relatives are highly under-represented in the global conservation system of plant genetic resources and may harbor important genes for resistance or tolerance to biotic and abiotic stresses.

## Taxonomy, wild relatives, and domestication of eggplant

Eggplants are berry-producing vegetables belonging to the large Solanaceae family (nightshade family), which contains ~3,000 species distributed in some 90 genera (Vorontsova and Knapp, 2012). Out of these *Solanum* L. is the largest one, with around 1,500 species (Frodin, 2004) including globally important crops such as potato (*Solanum tuberosum* L.) and tomato (*Solanum lycopersicum* L.), as well as many other minor crops. Most taxa of *Solanum* genus have a basic chromosome number of *n* = 12 (Chiarini et al., 2010).

**Figure 1:**
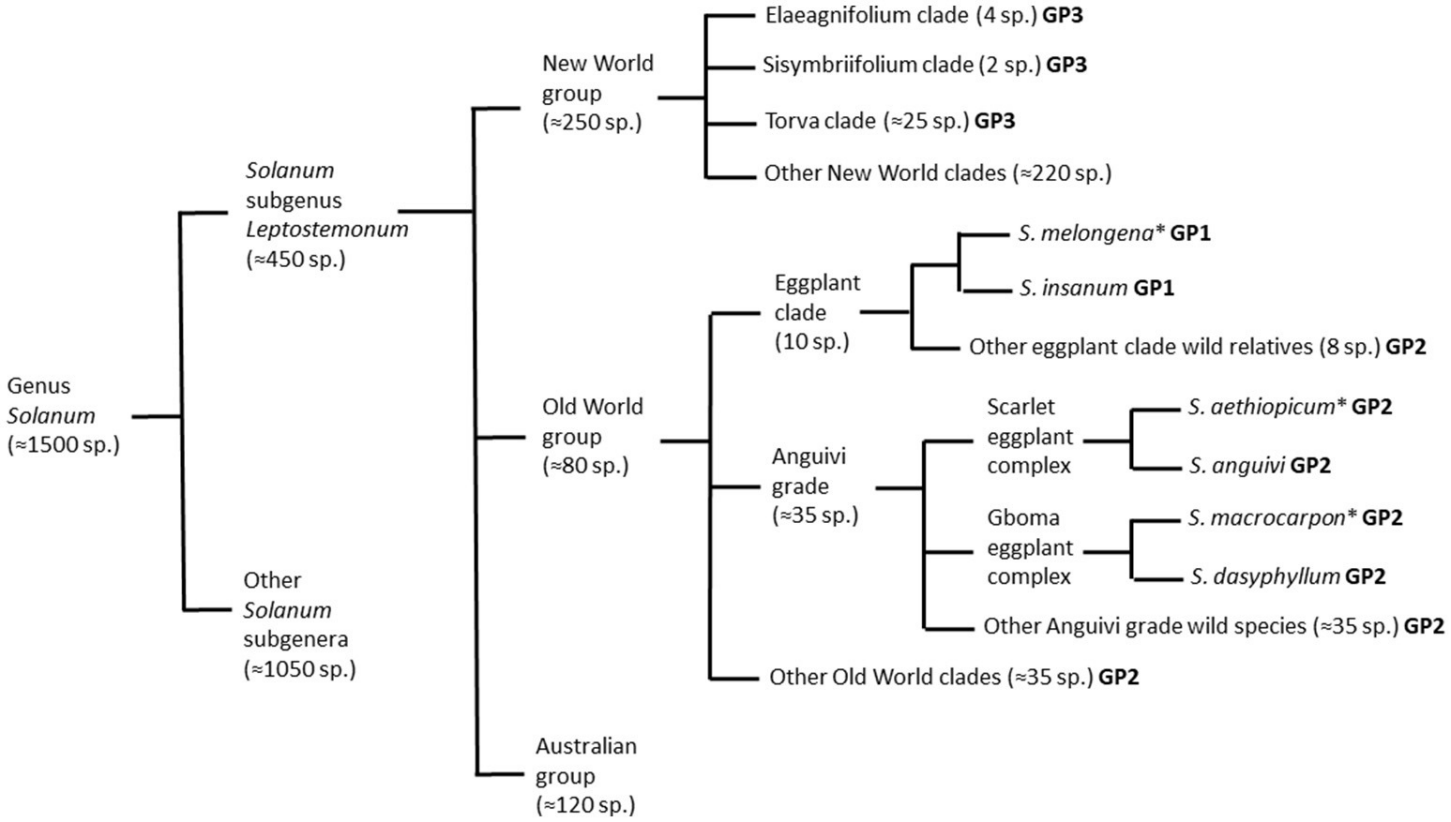
Schematic representation of taxonomic relationships between the cultivated brinjal eggplant (*Solanum melongena*) and other cultivated (scarlet eggplant, *S. aethiopicum*; and gboma eggplant, *S. macrocarpon*) and wild relatives from the genus *Solanum* based on Nee (1999), Levin et al. (2006), Weese and Bohs (2010), Stern et al. (2011), Knapp et al. (2013), Syfert et al. (2016), and Vorontsova and Knapp (2016). For each of the species and groups it is indicated if they are part of the primary (GP1), secondary (GP2), or tertiary (GP3) brinjal eggplant genepools. The three cultivated species are indicated with an asterisk.

The *Solanum* genus is mega-diverse and can be divided into 13 clades, where eggplant is the member of the large and taxonomically challenging *Leptostemonum* clade (subgenus *Leptostemonum* Bitter; Knapp et al., 2013), which is commonly known as the “spiny *Solanum*” group due to the presence of sharp epidermal prickles on stems and leaves (Vorontsova et al., 2013). The subgenus *Leptostemonum* contains around 450 currently recognized species distributed worldwide (Knapp et al., 2013), many of which originated in the New World (Vorontsova and Knapp, 2012). All three cultivated eggplant species have the Old World in origin (Figure 1). The Old World (Africa and Eurasia) and Australia, are home to more than 300 *Solanum* species (Levin et al., 2006; Vorontsova and Knapp, 2016). *Solanum melongena* and *S. macrocarpon* are usually included in section *Melongena* Dunal (Lester and Daunay, 2003; Lester et al., 2011), whereas *S. aethiopicum* is assigned to section *Oliganthes* (Dunal) Bitter (Lester, 1986).

*Solanum melongena* is characterized by large morphological diversity, and frequently it has been considered as the same taxonomic species than its wild ancestor *S. insanum* L. (Ranil et al., 2017). Four taxonomically informal groups, labeled E–H, were considered by Lester and Hasan (1991) to describe the different types of wild and weedy eggplant as well as their distribution (Table 1). However, these four groups are presently considered as representing two different species: the cultivated eggplant *S. melongena* and its wild ancestor *S. insanum* (Knapp et al., 2013). In this way, groups E and F corresponding to extremely prickly and plants that grow wild or weedy in India and Southeast Asia are now included within *S. insanum* (Ranil et al., 2017). The plants of group G correspond to primitive eggplant cultivars, with small fruits, while the plants of group H are less prickly than other groups and consist of large-fruited landraces and modern cultivars (Daunay et al., 2001; Weese and Bohs, 2010; Table 1). Both groups, G and H, constitute *S. melongena* (Knapp et al., 2013). Some studies (Hurtado et al., 2012; Vilanova et al., 2012; Cericola et al., 2013) have also pointed to a genetic and morphological differentiation between Occidental (eggplants from the Mediterranean area, North of Africa, and Middle East) and Oriental (from southeast and eastern Asia).

**Table 1:** Cultivated eggplants (brinjal eggplant, *S. melongena* L.; scarlet eggplant, *S. anguivi* L.; gboma eggplant, *S. macrocarpon* L.) and their wild relatives from the primary genepool, which correspond to their wild ancestors (*S. insanum* L. for brinjal eggplant, *S. anguivi* for scarlet eggplant, and *S. dasyphyllum* for gboma eggplant) (Lester, 1986; Lester and Niakan, 1986; Bukenya and Carasco, 1994; Schippers, 2000; Daunay et al., 2001; Weese and Bohs, 2010; Meyer et al., 2012; Knapp et al., 2013; Vorontsova and Knapp, 2016).

**Species**	**Groups**	**Form of occurrence**	**Fruit diameter (cm)**	**Prickliness**	**Bitterness**
**BRINJAL EGGPLANT COMPLEX**					
*S. melongena* L.	G	Cultivated (fruits)	3–4	Moderate	None to moderate
	H	Cultivated (fruits)	5–12	None to slight	None to slight
*S. insanum* L.	E	Weedy	1.5–2.5	Very high	Slight to moderate
	F	Weedy, wild	2–3	Moderate to high	Slight to moderate
**SCARLET EGGPLANT COMPLEX**					
*S. aethiopicum* L.	Aculeatum	Cultivated (ornamental)	3–8	High	Moderate
	Gilo	Cultivated (fruits)	2–10	None to slight	None to moderate
	Kumba	Cultivated (fruits and leaves)	5–10	None	None to slight
	Shum	Cultivated (leaves)	1.5–2.5	None	Moderate to high
*S. anguivi* L.	—	Wild, weedy	1–2	None to slight	High to very bitter
**GBOMA EGGPLANT COMPLEX**					
*S. macrocarpon* L.	Fruity	Cultivated (fruits)	5–12	None to slight	Slight to moderate
	Leafy	Cultivated (leaves)	2–6	None to slight	Slight to moderate
*S. dasyphyllum* Schumach. and Thonn.	—	Wild, weedy	3–4	Moderate to high	Moderate to high

*Solanum aethiopicum* is also hyper-variable and is classified into four cultivar groups (Gilo, Shum, Kumba, and Aculeatum; Table 1) based on morphological characteristics and use (Lester, 1986). The Gilo group has edible fruits with different shapes, color, and size, and hairy, inedible leaves; the Shum group has glabrous and small leaves that are eaten as a green vegetable but the fruits are inedible; the Kumba group has glabrous leaves and flattened large fruits, which are edible; the Aculeatum group, on the other hand, has more prickliness than other groups with flat-shaped fruit, and are used as ornamentals (Lester, 1986; Prohens et al., 2012; Plazas et al., 2014a).

*Solanum macrocarpon* is cultivated both for its leaves and fruits (Schippers, 2000; Maundu et al., 2009; Table 1). The species is less morphologically diverse than *S. melongena* and *S. aethiopicum* (Plazas et al., 2014a).

Although, recent information exists on domestication of eggplants, there are still many unanswered questions about this process. Vavilov (1951) considered *S. melongena* as being native to the “Indo-Chinese center of origin.” However, recent evidence suggests that brinjal eggplant had a multiple independent domestication from *S. insanum*, which is naturally distributed in tropical Asia from Madagascar to the Philippines (Knapp et al., 2013) in several centers of domestication (Meyer et al., 2012). Although, the evidence of cultivation of eggplant in both India and China is equally old, archeological evidence suggests that utilization of wild eggplants may have started earlier in India than China, with a subsequent additional and independent center of domestication in the Philippines (Meyer et al., 2012). Around the eighth century, eggplant spread eastward to Japan and then westward along the Silk Road into Western Asia, Europe, and Africa by Arab traders during the fourteenth century, then it was introduced into America soon after Europeans arrived there (Prohens et al., 2005) and later expanded into other parts of world. Much less is known on the domestication of the scarlet and gboma eggplants. Both species were domesticated in Africa, from its respective wild ancestors, which are *S. anguivi* Lam. in the case of *S. aethiopicum* (Lester and Niakan, 1986) and *S. dasyphyllum* Schumach. and Thonn. in the case of *S. macrocarpon* (Bukenya and Carasco, 1994). Hybrids between cultivated eggplants and their respective wild ancestors are fully fertile (Lester and Thitai, 1989; Bukenya and Carasco, 1994; Plazas et al., 2016).

*Solanum melongena* and the two other cultivated eggplants are related to a large number of wild species (Vorontsova et al., 2013; Syfert et al., 2016) that may serve as sources of variation for breeding programs, in particular for traits related to adaptation to climate change but also pest and disease resistance (Rotino et al., 2014). Some of these species are listed in Table 2. Although, the brinjal eggplant is considered to be a vegetable of Asian origin, most eggplant wild relatives are from Africa (Weese and Bohs, 2010). Wild eggplants produce small, bitter, multi-seeded fruits, almost always inedible, and the plant is generally very spiny. Some of them possess high levels of chlorogenic acid and other bioactive compounds, which may have potential interest for human health (Meyer et al., 2015). The wild relatives of eggplant are one of the most variable and intricate groups, in regards to their taxonomic and phylogenetic relationships (Vorontsova et al., 2013). Based on crossing and biosystematics data, nine wild species, together with *S. melongena*, form the “eggplant complex,” which includes the cultivated brinjal eggplant and its closest eggplant wild relatives (Knapp et al., 2013). Wild relatives can be classified based on their crossability with cultivated species (genepool concept) into primary, secondary, and tertiary genepools (Harlan and de Wet, 1971). The primary genepool (GP1) of brinjal eggplant consists of cultivated eggplant and its wild ancestor *S. insanum* (Ranil et al., 2017) which can be crossed easily and produce normal fertile hybrids (Plazas et al., 2016). The secondary genepool (GP2) includes a large number (over 40) wild relatives that can be crossed or are phylogenetically close to brinjal eggplant, but the success of the crosses and the viability or fertility of the hybrids with the brinjal eggplant may be reduced. For example, some interspecific hybrids derived from GP2 are partly sterile or weak due to reproductive barriers such as *S. dasyphyllum, S. linnaeanum* Hepper & P.-M. L. Jaeger or *S. tomentosum* L. (Rotino et al., 2014; Kouassi et al., 2016). The tertiary genepool (GP3) includes more distantly related species, including New World species, which are used in breeding programs for their resistance features, but crossing needs specific breeding techniques to succeed (e.g., *S. torvum* Sw., *S. elaeagnifolium* Cav., and *S. sisymbriifolium* Lam.; Kouassi et al., 2016; Plazas et al., 2016; Syfert et al., 2016).

**Table 2:** Cultivated eggplant and wild relatives, number of occurrences, their regions and number of conserved accessions globally and at the World Vegetable Center (WorldVeg).

**Scientific name**	**Global occurrences (GBIF, 2017)**		**Genebank holdings (AVGRIS, 2017; GENESYS, 2017)**			
	**Number of records**	**Clusters of occurrences**	**Global number of accessions**	**WorldVeg number of accessions**	**% WorldVeg of global**	**Largest collection**
Cultivated eggplant	19,999		6,632	2,756	42	
*Solanum melongena* L.	18,268	India, W&SE Asia, Spain	5,665	2,212	39	WorldVeg
*S. aethiopicum* L.	1,288	W Africa	798	481	60	WorldVeg
*S. macrocarpon* L.	443	W Africa	169	63	37	WorldVeg
Wild relatives of eggplant	55,414		1,304	418	32	
*S. aculeatissimum* Jacq.	1,506	E Africa, China, Brazil	65	46	71	WorldVeg
*S. anguivi* L.	2,739	T Africa	83	23	28	WorldVeg
*S. atropurpureum* Schrank	718	Brazil	21	1	5	Radboud University
*S. aviculare* G. Forst.	1,947	New Zealand, E Australia	25	2	8	Radboud University
*S. campylacanthum* Hochst. ex A.Rich.	1,253	E Africa	10	1	10	University of Nijmegen
*S. capense* L.	585	S Africa	8	3	38	WorldVeg
*S. capsicoides* All.	1,916	L America	30	2	7	Radboud University
*S. dasyphyllum* Schumach. and Thonn.	495	T Africa	21	3	14	Millennium Seed Bank
*S. elaeagnifolium* Cav.	5,891	N&L America	30	3	10	Millennium Seed Bank
*S. erianthum* D. Don	4,534	L America, SE Asia, E Australia	9	2	22	Millennium Seed Bank
*S. ferox* L.	128	SE Asia	29	11	38	WorldVeg
*S. incanum* L.	1,122	Africa	167	5	3	University of Nijmegen
*S. indicum L*.	227	E Asia	13	12	92	WorldVeg
*S. insanum L*.	290	E Asia	11	11	100	WorldVeg
*S. laciniatum* Aiton	1,459	New Zealand, E Australia, Europe	38	3	8	Radboud University
*S. lasiocarpum* Dunal	681	Oceania	42	31	74	WorldVeg
*S. linnaeanum* Hepper & P.-M.L. Jaeger	1,457	Spain, Africa, S Australia	48	3	6	Radboud University
*S. pectinatum* Dunal	246	L America	11	1	9	Radboud University
*S. pseudocapsicum* L.	4,938	L America	41	3	7	Radboud University
*S. quitoense* Lam.	803	L America	63	1	2	University of Nijmegen
*S. repandum* G. Forst.	111	No information	4	1	25	
*S. rostratum* Dunal	3,615	N America	27	1	4	Millennium Seed Bank
*S. seaforthianum* Andrews	2,266	L America	14	3	21	WorldVeg
*S. supinum* Dunal	425	S Africa	5	1	20	University of Nijmegen
*S. sessiliflorum* Dunal	604	L America	19	1	5	Radboud University
*S. sisymbriifolium* Lam.	3,466	L America	88	19	22	Radboud University
*S. stramoniifolium* Jacq.	1,394	L America	16	10	63	WorldVeg
*S. torvum* Sw.	7,379	L America, W Africa, SE Asia	132	112	85	WorldVeg
*S. trilobatum* L.	257	SE Asia	14	10	71	WorldVeg
*S. viarum* Dunal	1,063	L America	59	16	27	WorldVeg
*S. violaceum* Ortega	1,149	SE Asia	64	49	77	WorldVeg
*S. virginianum* L.	633	SE Asia	31	3	10	Millennium Seed Bank
*S. xanthocarpum* Schrad. & J.C.Wendl.	117	E Asia	20	18	90	WorldVeg
All species globally (grand total)	703,244,524		3,611,454	61,982	1.7	

## Global occurrences and genebank conservation of eggplant and wild relatives

In the following section we review the current status of eggplant genetic resources including the cultivated species and their most recognized wild relatives using information collected from biodiversity, herbarium, and genebank databases. The Global Biodiversity Information Facility (GBIF) was applied to review the number of recorded occurrences, which can be natural populations, herbarium samples, or other biodiversity records (GBIF, 2017). Scientific names were used as a filter in the search function. The total numbers of records per species were noted, as were clusters of occurrences that were identified visually by applying the database map function. The main cluster of *S. melongena* was in India, with more than 5,000 of the total number of around 18,000 occurrences. Other clusters were in Turkey, Southeast Asia, and Spain, while the main cluster of occurrences of *S. aethiopicum* and *S. macrocarpon* was in West Africa, with a total of 1,288 and 443 occurrences, respectively. Based on the literature of previous studies and characterization data available at the WorldVeg, a list of 35 crop wild relatives was included in this review, which had ~100 (*S. repandum* G. Forst.) to more than 7,000 occurrences (*S. torvum*) on a global scale recorded by GBIF (Table 2). Important regions for wild relatives vary depending on the species, but include all continents; Latin America, Asia, and Africa are the most common areas for wild relatives.

The Global Gateway to Genetic Resources (GENESYS, 2017) was applied to review the number of conserved genebank accessions. The database includes more than 3 million accessions, which is less than half of the estimated number of more than 7 million accessions that are conserved globally (FAO, 2010). Although, not all national genebanks report to Genesys, we still used the information for reviewing global holdings. Scientific names were used as a filter in the search function of the database, and the most important holding institutions were identified from the summary function of the database. Additional sources were reviewed to try to capture important collections outside Genesys, including national genebank databases and the database for Svalbard Global Seed Vault (SGSV, 2017). The WorldVeg plays a major role in the conservation and distribution of vegetable germplasm, holding 60,387 accessions comprising 173 genera and 440 species from 151 countries of origin (AVGRIS, 2017).

In total, 5,665 accessions of *S. melongena*, 798 accessions of *S. aethiopicum* and 169 accessions of *S. macrocarpon* were reported by GENESYS (2017). Important national eggplant collections not reporting to GENESYS are at the National Bureau of Plant Genetic Resources in India and the Institute of Vegetables and Flowers in China. Data from such collections were not included in our study. The largest collections of these three cultivated species were those of the WorldVeg [2,212 accessions of *S. melongena* (39%), 481 accessions of *S. aethiopicum* (60%), and 63 accessions of *S. macrocarpon* (37%)], followed by the Plant Genetic Resources Conservation Unit at the University of Georgia, USDA-ARS (close to 800 accessions of *S. melongena*) and the Centre for Genetic Resources at the Netherlands Plant Research International (373 accessions of *S. melongena*; GENESYS, 2017). The N. I. Vavilov Research Institute of Plant Genetic Resource in Russia has a significant eggplant collection with more than 500 *S. melongena* accessions. The conservation of wild species ranged from a few accessions (e.g., *S. rigescentoides* Hutch.) to 167 accessions (*S. incanum* L.). None of the wild species had large collections. Interestingly, the WorldVeg has the largest collections for *S. aculeatissimum* Jacq. (46 accessions, 71%), *S. anguivi* (28 accessions, 23%), *S. capense* L. (3 accessions, 38%), *S. ferox* L. (11 accessions, 38%), *S. indicum* L. (12 accessions, 92%), *S. insanum* (11 accessions, 100%), *S. lasiocarpum* Dunal (31 accessions, 74%), *S. stramoniifolium* Jacq. (10 accessions, 63%), *S. torvum* (112 accessions, 85%), *S. trilobatum* L. (10 accessions, 71%), *S. viarum* Dunal (16 accessions, 27%), *S. violaceum* Ortega (49 accessions, 77%), and *S. xanthocarpum* Schrad. & J. C. Wendl. (18 accessions, 90%) (GENESYS, 2017). The low number of accessions identified as *S. insanum* in the collections is surprising, taking into account that it is quite abundant and the progenitor of eggplant (Knapp et al., 2013; Ranil et al., 2017). This is probably caused by the fact that many *S. insanum* accessions are probably conserved as *S. melongena*, as both species have often been considered as being a single species (*S. melongena*; e.g., Lester and Hasan, 1991). Also, the correct classification of accessions under “*S. indicum* L.” should be determined, as this name was rejected in 1978 as it was used to refer to two clearly distinct species, the African *S. anguivi* and the Asian *S. violaceum* (Vorontsova and Knapp, 2016).

According to our analysis, wild eggplants are greatly under-represented in *ex situ* repositories. Such findings are also reported by Castañeda-Álvarez et al. (2016), where eggplants were among the crops whose wild genepools are highly under-represented. Indeed, there is a need for conducting collection missions and conservation actions for eggplant wild relatives (Conservation gaps, http://www.cwrdiversity.org/conservation-gaps/, Accessed February 30, 2017).

## Eggplant germplasm dissemination from the world vegetable center

As demonstrated in the previous section, the collection at the WorldVeg is the most significant eggplant collection worldwide. Eggplant is the Center's third most widely distributed vegetable crop after pepper and tomato. A total of 11,383 germplasm samples were distributed from WorldVeg headquarters to 90 countries from the period 2000 to 2017. Most of these were of *S. melongena* (10,519 samples; 92.4%), followed by *S. aethiopicum* (738 samples; 6.4%) and *S. macrocarpon* (126 samples; 2.2%; Table 3). These accessions correspond to landraces and traditional cultivars with significant diversity in plant morphology, fruit types and colors, and resistance to biotic and abiotic stresses. The largest share of germplasm samples went to other genebanks (7,042 samples; 61.8%), followed by National Agricultural Research & Extension System/Government (NARES) (2,154 samples; 18.9%), internal distribution to WorldVeg scientists (703 samples; 6.1%), and seed companies (503 samples; 4.4%).

**Table 3:** The World Vegetable Center seed distribution of cultivated eggplant by recipient category during the period 2000–2017.

**Recipient category**	**Number of seed samples**			
	***S. melongena***	***S. macrocarpon***	***S. aethiopicum***	**Total**
**INTERNAL**				
WorldVeg Headquarters	564	7	132	703
WorldVeg Regional Offices	181	2	87	270
**EXTERNAL**				
Other genebanks^*^	6,607	94	341	7,042
National Agricultural Research & Extension Systems	2,046	17	91	2,154
Universities	418	2	14	434
Seed companies	452	1	50	503
Other companies	49	–	4	53
Non-government organization	80	2	18	100
Individuals	122	1	1	124
Total	10,519	126	738	11,383

* *Including back-up of accessions in other genebanks*.

The large morphological diversity of the WorldVeg collection is matched by the identification of traits of significant agronomic interest. WorldVeg has compiled and maintained the world's largest germplasm collection of eggplant, and national genebanks and institutions from around the globe have requested and received many samples. A significant number of accessions are internal distributions to WorldVeg regional offices, and in collaboration with partner institutions, the material has been used in breeding programs. New open-pollinated varieties have been released in Uzbekistan, Tanzania, and Mali through selection based on local trait preferences (Table 4).

**Table 4:** List of eggplant and African eggplant varieties released in Uzbekistan, Tanzania, and Mali based on WorldVeg germplasm.

**Crop**	**Locally released Commercial name**	**WorldVeg code**	**Country**	**Year released**	**Salient known features recorded in the country where released**
Eggplant	Tukhfa	VI034954 or S00113	Uzbekistan	2016	Mid-maturing variety, 130 days, bush type, semi-spreading. Yield around 25 t/ha. Resistant to *Fusarium*. Fruit weight around 117 g, elongate-cylindrical fruits, curved, light purple skin, flesh is bright and tender.
Eggplant	Kuvonch	VI042717	Uzbekistan	2015	Mid-maturing variety, 130 days, bush type, semi-spreading. Yield around 26 t/ha, Resistant to *Fusarium*. Fruit weight is 140 g. Fruits are oblong-cylindrical, dark violet color. Fruits are transportable.
Eggplant	Feruz	VI042320	Uzbekistan	2013	Large, elliptical-shaped fruits (180 g) and yields 32 t/ha (Mavlyanova 2015).
African eggplant	Mshumaa	DB 3	Tanzania	2011	DB3 is sweet, has nearly replaced the bitter-tasting landraces.
African eggplant	Soxna		Mali	2011	High yielding, small sized fruits with a slightly bitter taste preferred by consumers.
African eggplant	L10		Mali	2011	High yielding with a slightly bitter taste preferred by consumers.

## Utilization of eggplant germplasm in breeding

Screening of available accessions for targeted traits (evaluation) and morphological description of the accessions (characterization) are key issues for the breeding process. At the WorldVeg a large number of commercial cultivars, landraces, and germplasm have thus been examined to identify desired genotypes for use in eggplant breeding programs or for recommending to private sector seed companies and other partner institutions. Standardized descriptors included characters both for germination, the vegetative phase, inflorescence descriptors, and fruit and seed traits, respectively (Table 5).

**Table 5:** A complete list of standard descriptors for eggplants used at the World Vegetable Center (AVGRIS, 2017).

**Code**	**Definition**	**Scale**
S110	Germination period	Number of days from sowing until first germination
S120	Cotyledonous leaf length	mm (*N* = 10)
S130	Cotyledonous leaf width	mm (*N* = 10)
S140	Cotyledonous leaf color	3 = Green, 5 = Light violet, 7 = Violet, X = Mixture
S150	Cotyledon length/width ratio	1 = Very low (<2.0), 3 = Low (~2.2), 5 = Intermediate (~2.5), 7 = High (~3.5), 9 = Very high (>5.0), X = Mixture
S210	Plant growth habit	1 = Very upright, 3 = Upright, 5 = Intermediate, 7 = Prostrate, X = Mixture
S215	Stem ridging	0 = Absent, 3 = Shallow, 5 = Intermediate, 7 = Prominent, X = Mixture
S216	Prickles on stem	0 = Absent, 3 = Short, 5 = Intermediate, 7 = Long, X = Mixture
S217	Degree of stem pubescence	0 = Absent, 1 = Few, 2 = Intermediate, 3 = Many, 4 = Very many, X = Mixture
S220	Plant height at flowering (cm)	1 = Very short (<20), 3 = Short (~30), 5 = Intermediate (~60), 7 = Tall (~100), 9 = Very tall (>150), X = Mixture
S230	Plant branching (no. of primary branches per plant)	1 = Very weak (~2), 3 = Weak (~5), 5 = Intermediate (~10), 7 = Strong (~20), 9 = Very strong (>30), X = Mixture, *M* = Uncountable
S240	Plant breadth at flowering (cm)	1 = Very weak (~2), 3 = Weak (~5), 5 = Intermediate (~10), 7 = Strong (~20), 9 = Very strong (>30), X = Mixture, *M* = Uncountable
S250	Petiole color	1 = Green, 2 = Greenish violet, 3 = Violet, 7 = Dark violet, 9 = Dark brown, X = Mixture
S260	Petiole length (mm)	0 = None, 1 = Very short (<5), 3 = Short (~10), 5 = Intermediate (~30), 7 = Long (~50), 9 = Very long (>100), X = Mixture
S270	Leaf blade length (cm)	3 = Short (~10), 5 = Intermediate (~20), 7 = Long (~30), X = Mixture
S280	Leaf blade width (cm) (maximum width)	3 = Narrow (~5), 5 = Intermediate (~10), 7 = Wide (~15), X = Mixture
S290	Leaf blade lobes	1 = Very weak, 3 = Weak, 5 = Intermediate, 7 = Strong, 9 = Very strong, X = Mixture
S300	Leaf blade tip angle (°)	1 = Very acute (<15°), 3 = Acute (~45°), 5 = Intermediate (~75°), 7 = Obtuse (~110°), 9 = Very obtuse (>160°), X = Mixture
S310	Leaf blade color (upper surface)	1 = Light green, 3 = Green, 5 = Dark green, 7 = Greenish violet, 9 = Violet, X = Mixture
S320	Leaf prickles (no. of leaf prickles on upper surface of the leaf)	0 = None, 1 = Very few (1–2), 3 = Few (3–5), 5 = Intermediate (6–10), 7 = Many (11–20), 9 = Very many (>20), X = Mixture
S330	Leaf hairs (no./per mm^2^, lower surface)	1 = Very few (<20), 3 = Few (20–50), 5 = Intermediate (50–100), 7 = Many (100–200), 9 = Very many (>200), X = Mixture
S410	Flowers per inflorescence	Number (*N* = 10)
S420	Flowering time	Number of days from sowing until first flower opening (*N* = 10)
S421	Stamen length	cm (*N* = 5)
S422	Petal length	cm (*N* = 5)
S423	Sepal length	cm (*N* = 5)
S430	Number of hermaphrodite flowers per inflorescence	1 = One, 2 = Two, 3 = Three, 4 = Four or more but some flowers functionally male, 5 = Four or more, no functionally male, X = Mixture
S440	Corolla color	0 = Yellow, 1 = Greenish white, 3 = White, 5 = Pale violet, 7 = Light violet, 9 = Bluish violet, X = Mixture
S450	Relative style length (mm)	3 = Short (~1), 5 = Intermediate (~3), 7 = Long (~5), X = Mixture
S460	Pollen production	0 = None, 3 = Low, 5 = Medium, 7 = High, X = Mixture
S470	Style exsertion	3 = Inserted, 5 = Intermediate, 7 = Exerted, X = Mixture
S510	Fruit length from base of calyx to tip of fruit (cm)	1 = Very short (<1), 3 = Short (~2), 5 = Intermediate (~5), 7 = Long (~10), 9 = Very long (>20), X = Mixture
S520	Fruit breadth diameter at broadest part (cm)	1 = Very small (<1), 3 = Small (~2), 5 = Intermediate (~3), 7 = Large (~5), 9 = Very large (>10), X = Mixture
S530	Fruit length/breadth ratio	1 = Broader than long, 3 = As long as broad, 5 = Slightly longer than broad, 7 = Twice as long as broad, 8 = Three times as long as broad, 9 = Several times as long as broad, X = Mixture
S540	Fruit curvature	1 = None, 3 = Slightly curved, 5 = Curved, 7 = Snake shaped, 8 = Sickle shaped, 9 = U shaped, X = Mixture
S550	Fruit pedicel length (mm)	1 = Very short (<5), 3 = Short (~10), 5 = Intermediate (~25), 7 = Long (~50), 9 = Very long (~75), X = Mixture
S560	Fruit pedicel thickness (mm)	1 = Very thin (<1), 3 = Thin (~2), 5 = Intermediate (~3), 7 = Thick (~5), 9 = Very thick (>10), X = Mixture
S570	Fruit pedicel prickles	0 = None, 1 = Very few (<3), 3 = Few (~5), 5 = Intermediate (~10), 7 = Many (~20), 9 = Very many (>30), X = Mixture
S580	Fruit shape	3 = About 1/4 way from base to tip, 5 = About 1/2 way from base to tip, 7 = About 3/4 way from base to tip, X = Mixture
S590	Fruit apex shape	3 = Protruded, 5 = Rounded, 7 = Depressed, X = Mixture
S600	Fruit color at commercial ripeness	1 = Green, 2 = Milk white, 3 = Deep yellow, 4 = Fire red, 5 = Scarlet red, 6 = Lilac gray, 7 = Purple, 8 = Purple black, 9 = Black, X = Mixture
S610	Fruit color distribution at commercial ripeness	1 = Uniform, 3 = Mottled, 5 = Netted, 7 = Striped, X = Mixture
S620	Fruit color at physiological ripeness	1 = Green, 2 = Deep yellow, 3 = Yellow orange, 4 = Deep orange, 5 = Fired red, 6 = Poppy red, 7 = Scarlet red, 8 = Light brown, 9 = Black, X = Mixture
S630	Fruit position	1 = Erect, 3 = Semi-erect, 5 = Horizontal, 7 = Semi-pendant, 9 = Pendant, X = Mixture
S640	Relative fruit calyx length	mm (*N* = 10)
S650	Fruit calyx prickles (N = 10)	0 = None, 1 = Very few (<3), 3 = Few (~5), 5 = Intermediate (~10), 7 = Many (~20), 9 = Very many (>30), X = Mixture
S660	Fruit cross section	1 = Circular, no grooves, 3 = Elliptic, no grooves, 5 = Few grooves (~4), 7 = Many grooves (~8), 9 = Very irregular, X = Mixture
S680	Locules per fruit	Number (*N* = 10)
S690	Fruit flesh density	1 = Very loose (spongy), 3 = Loose (crumbly), 5 = Average density, 7 = Dense, 9 = Very dense, X = Mixture
S700	Fruits per infructescence	Number (*N* = 10)
S710	Fruit per plant	Number (*M* = Uncountable)
S720	Fruit yield per plant (gm)	1 = Very low (<250), 3 = Low (~500), 5 = Intermediate (~1,000), 7 = High (~2,500), 9 = Very high (>5,000), X = Mixture
S730	Fruit flavor	3 = Bitter, 5 = Intermediate, 7 = Sweet, X = Mixture
S760	Varietal mixture condition	0 = Pure, 3 = Slight mixture, 5 = Medium mixture, 7 = Serious mixture
S770	Flesh browning after cutting	1 = 0–1 min, 2 = 1–3 min, 3 = 3–5 min, 4 = 5–7 min, 5 = 7–9 min, 6 = 9–12 min, 7 = 12–15 min, 8 = 15–20 min, 9 = 20–30 min, 10 = 30 min or more
S810	Seed color	1 = White, 2 = Light yellow, 3 = Grey yellow, 4 = Brownish yellow, 5 = Brown, 6 = Brown black, 9 = Black, X = Mixture
S820	Seeds per fruit	0 = None, 1 = Very few (<10), 3 = Few (~50), 5 = Intermediate (~100), 7 = Many (~300), 9 = Very many (>500), X = Mixture
S825	Seed density	3 = Scarce, 5 = Intermediate, 7 = Dense, X = Mixture
S830	Seed size (mm)	3 = Small (~2), 5 = Intermediate (~3), 7 = Large (~4), X = Mixture
S840	100 seeds weight	g (average of 3 replicates)
S900	Harvest produce	1 = Bulk, 2 = 2 sub-accessions, 3 = 3 sub-accessions

**Figure 2:**
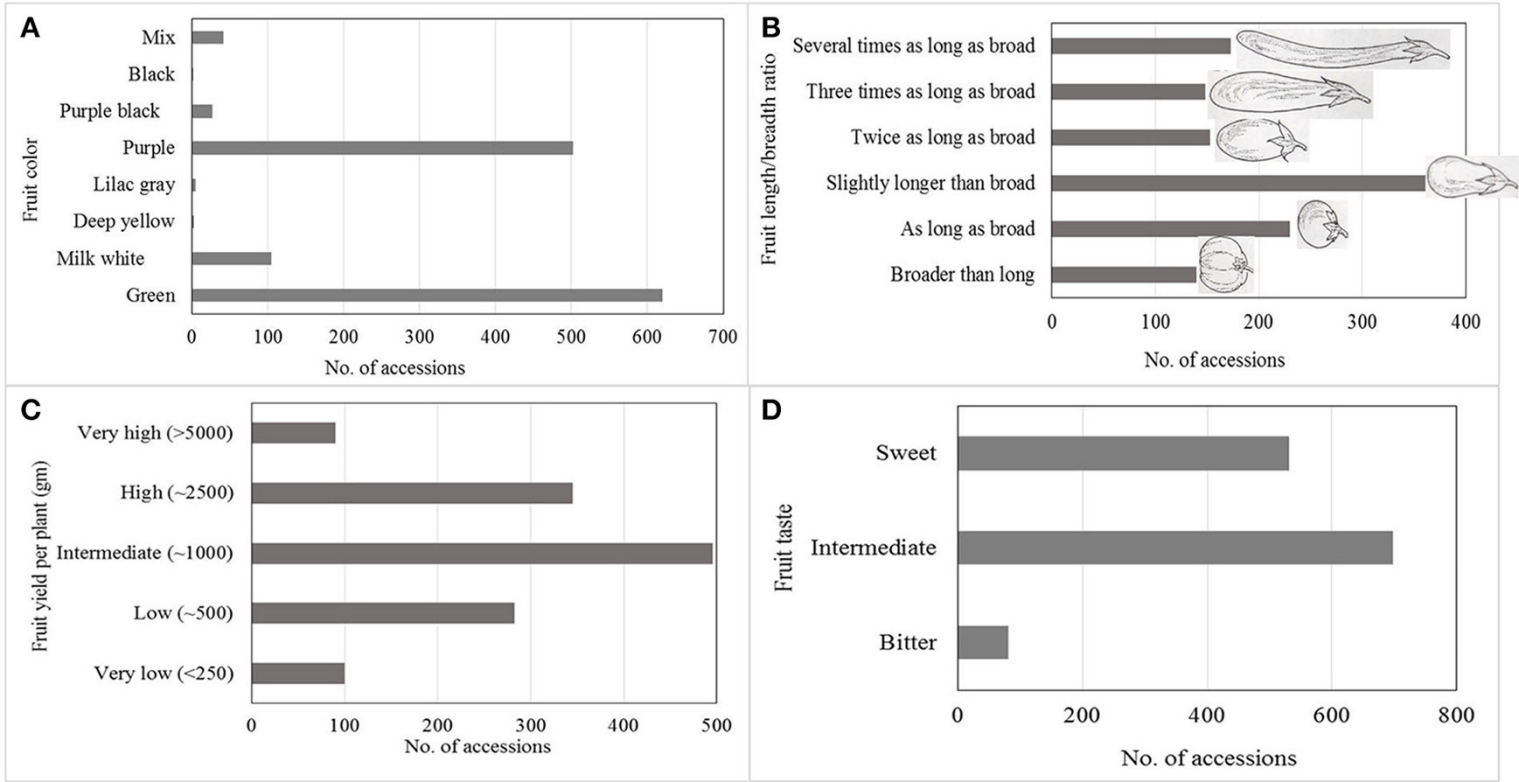
Horticultural characteristics of more than 1,300 accessions of *Solanum melongena* summarized and based on information available in AVGRIS (2017): **(A)** Fruit color, **(B)** Fruit length, **(C)** Fruit yield per plant, and **(D)** Fruit taste.

**Figure 3:**
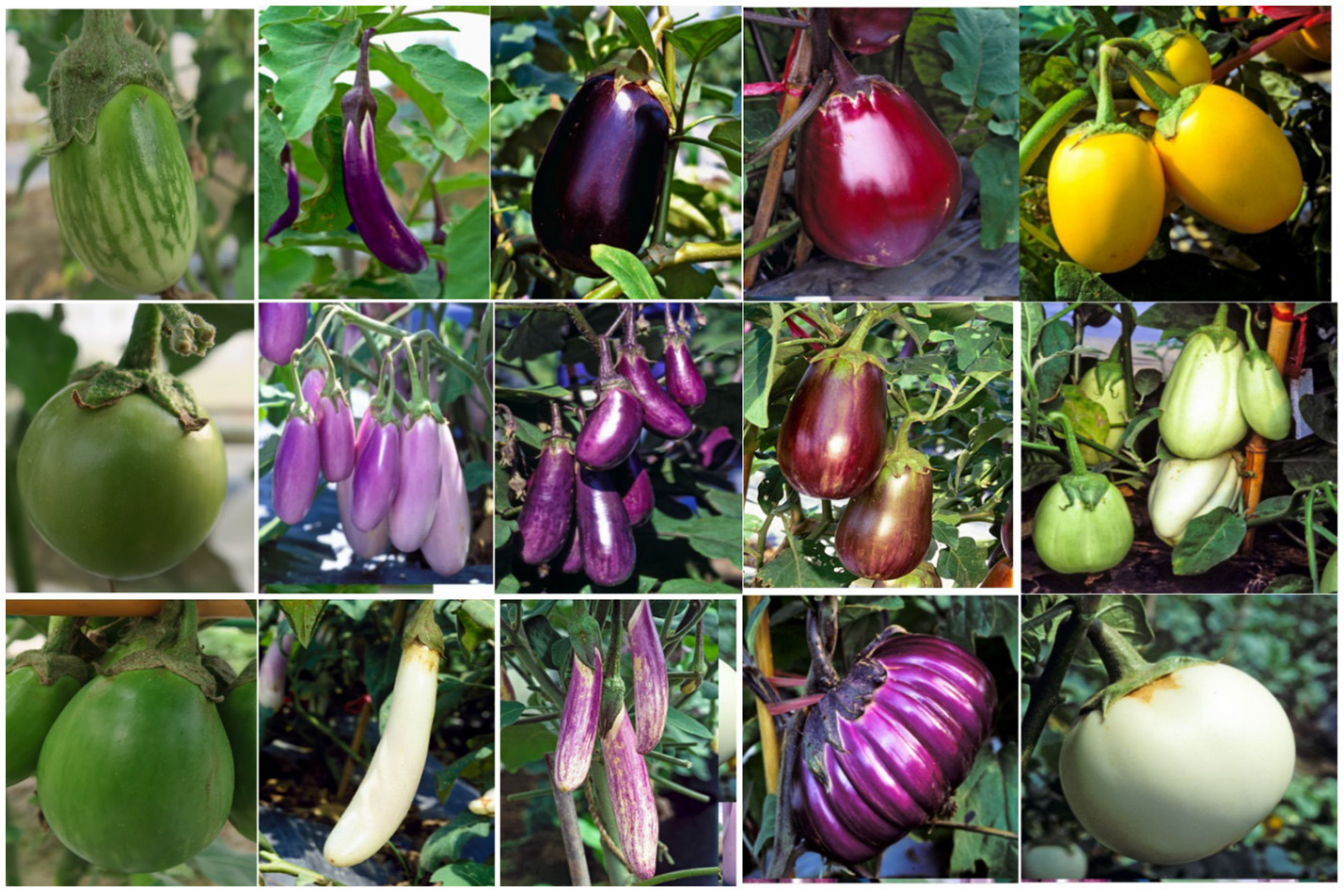
Different fruit shapes, colors, and sizes of *Solanum melongena* accessions in the World Vegetable Center germplasm collection.

Large variation in yield parameters and in fruit quality parameters have been documented in the collection (Figures 2, 3). Such data have been compiled over years and can be retrieved from AVGRIS, the World Vegetable Center genebank database system (2017). Among the 1,308 accessions of *S. melongena* that have been characterized, green and purple fruits were predominant, and could be found in 47 and 38% of the total number of accessions, respectively. Slightly longer than broad, and as long as broad, were the prevalent shapes of the accessions, with 31.1 and 18.7%, respectively. Similarly, huge diversity was found among 98 accessions belong to *S. melongena, S. aethiopicum*, and *S. macrocarpon* for 16 morpho-agronomic and fruit traits including plant height, flowering time, flower/inflorescence, fruit length and fruit acidity, but weak association was found between among morpho-agronomic and fruit quality descriptors (Polignano et al., 2010). In terms of fruit taste, 26.8% of accessions had a sweet taste, 53.2% had an intermediate taste and some accessions had bitter taste (6.1%). Large variations in fruit dry matter content, total sugar content, and fiber content of the fruit have been determined in a study of 90 selected eggplant genotypes (AVRDC, 1996). The distribution of dry matter, total sugar, and fiber contents ranged from 5.5 to 10.1, 7.0 to 40.1, and 4.7 to 18.1%, respectively. In another study conducted at the WorldVeg, 33 *S. melongena* accessions and two *S. aethiopicum* accessions were evaluated for superoxide scavenging and content of total phenolics and ascorbic acid (Hanson et al., 2006). *Solanum melongena* accessions S00062, S00022, and *S. aethiopicum* accession S00197 exhibited high antioxidant activity (Hanson et al., 2006).

Accessions with important traits such as early maturity, high yielding, and resistance to biotic stresses have been identified in the WorldVeg germplasm collection (Table 6). Based on data from Chen (1998) and the examination of 40 accessions from the WorldVeg collection, among long fruit genotypes, VI045551, VI047333, VI046110, and VI037736 were identified as stable and high yielding (>40 tons per hectare) over spring, summer, and autumn seasons. Accession VI046110 had the highest average yield and the earliest maturing genotype across the three seasons (AVRDC, 1999). In round fruit type, VI046097, VI047332, VI44067, EG233, and EG235 produced the high yields in all three seasons.

**Table 6:** Identified eggplant germplasm from the World Vegetable Center collection with useful traits for breeding.

**Trait**	**Taxa and accessions code**	**References**
High yield	*S. melongena:* VI045551, VI047333, VI046110, VI037736, VI046097, VI047332, VI44067, EG233, and EG235	Chen, 1998
Early yield	*S. melongena*: VI046110	AVRDC, 1999
High antioxidant activity	*S. melongena*: S00062, S00022, and *S. aethiopicum*: S00197	Hanson et al., 2006
Resistance to bacterial wilt (*Ralstonia solanacearum*)	*S. melongena*: TS3, VI034885, TS47, TS69, TS87, and TS90	AVRDC, 1999
Resistance to leafhopper (*Amrasca devastans*) and aphids (*Aphis gossypii*)	*S. melongena*: VI034971, VI035822, and VI035835	AVRDC, 1999
Resistance to eggplant fruit and shoot borer (*Leucinodes orbonalis*)	*S. melongena*: VI047451	Ramasamy, 2009

Based on data from AVGRIS (2017) compiled over the years and including 1,300 accessions, only 90 accessions (6.8%) had more than 5,000 g of fruit yield per plant (Figure 2). Marketable yields were highly associated with fruit weight and number of fruits per plant. Large diversity in the WorldVeg germplasm collections enabled us to develop several improved eggplant and African eggplant cultivars (Table 3). A total of three eggplant varieties have been commercialized in Uzbekistan and three African eggplant varieties have been released in Tanzania and Mali.

More than 200 accessions have been evaluated for resistance to bacterial wilt (*Ralstonia solanacearum*) at the WorldVeg under both greenhouse and field conditions (AVRDC, 1999). Among these, 38 accessions were identified with high levels of resistance. These accessions were retested using root wounding and soil drenching inoculation methods in the greenhouse. Data were summarized from the screening and retest studies, and the most resistant accessions were TS3, VI034885, and TS47 from Malaysia; and TS69, TS87, and TS90 from Indonesia with disease indices <10% under both greenhouse and field conditions.

Resistance to eggplant fruit and shoot borer (*Leucinodes orbonalis* Guenee), leafhopper (*Amrasca devastans* Distant), and aphids (*Aphis gossypii* Glover) have been identified at WorldVeg in separate trials (AVRDC, 1999). Leafhoppers and aphids have piercing mouthparts and suck the sap, especially from the leaves, which leads to yellow spots on the leaves, followed by crinkling, curling, bronzing, and drying (or “hopper burn” from leafhopper), but severe aphid infestations cause young leaves to curl and become deformed (AVRDC, 1999; Ramasamy, 2009). Like whiteflies, aphids also produce honeydew, which leads to the development of sooty mold (Ramasamy, 2009). Accessions VI034971, VI035822, and VI035835 were found promising accessions against leafhopper and aphids. Eggplant fruit and shoot borer is an extremely destructive pest, especially in South and Southeast Asia (Ramasamy, 2009). It lays eggs on the foliage and neonate larvae feeds on the tender shoots, boring into the shoots and fruits, resulting in wilting of young shoots, followed by drying; the fruit becomes unfit for marketing and consumption. Total resistance was not found and moderate resistance was found only in one accession, VI047451 (AVRDC, 1999). This was based on typical damage symptoms, wilting of shoots and feeding holes in a wilted shoot, as well as damaged fruit. Overall, these results show that very promising materials for breeding pest tolerant or resistant varieties can be found in the WorldVeg eggplant collection. However, additional race specific screening is needed to find resistant sources for pests where no resistance or limited resistance has been found.

## The way forward

The food security of many countries relies on crops bred from genetic resources outside their region (Khoury et al., 2016). Therefore, plant genetic resources are a global concern where access and benefit sharing is of paramount importance. Eggplant is an important vegetable crop with a global cultivation area. From the current study we have confirmed that there are critical gaps in global eggplant collections, especially related to crop wild relatives (Syfert et al., 2016). We have listed more than 35 wild species conserved in germplasm collections, but for many other eggplant wild relatives no accessions are conserved in genebanks; in addition, there still might be undiscovered crop wild relatives. Genetic diversity in wild relatives is much higher than in cultivated eggplant (Vorontsova et al., 2013) and could be valuable sources for resistance to biotic and abiotic stresses (Daunay and Hazra, 2012). To date, a limited number of wild relatives have used in eggplant breeding (Rotino et al., 2014) and commercial varieties containing wild relative introgressions are not yet available. To move forward, screening for abiotic and biotic stresses in wild relatives should be intensified and broadened for identification of valuable germplasm accessions for breeding improved eggplant varieties. This information, combined with genomics studies for the detection of genes and QTLs of agronomic importance and their associated markers, will be of great utility in eggplant breeding, as has been demonstrated in some association mapping studies (Cericola et al., 2014; Portis et al., 2015). Recent reviews of the development in eggplant is provided by Frary and Doganlar (2013) and Gramazio et al. (in press).

From a utilization point of view, core collections could be established and stakeholders should work together for the development of the next generation of eggplant varieties that can meet the challenges of the present and the future.

## Author contributions

DT compiled the major parts of the text; SS contributed with text on genetic resources; JP contributed with text on eggplant wild relatives; YC contributed with reviewing databases; MR and TW contributed with inputs on eggplant taxonomy and breeding.

### Conflict of interest statement

The authors declare that the research was conducted in the absence of any commercial or financial relationships that could be construed as a potential conflict of interest.
